# Localized Soft Vibrational Modes and Coherent Structural Phase Transformations in Rutile TiO_2_ Nanoparticles under Negative Pressure

**DOI:** 10.1021/acs.nanolett.2c01939

**Published:** 2022-07-07

**Authors:** Kang Wang, Carla Molteni, Peter D. Haynes

**Affiliations:** † Imperial College London, Department of Materials, Exhibition Road, London SW7 2AZ, U.K.; ‡ King’s College London, Department of Physics, Strand, London WC2R 2LS, U.K.

**Keywords:** TiO_2_ nanoparticles, soft vibrational modes, localized distortion, size effect, coherent structural phase transformations, density functional theory

## Abstract

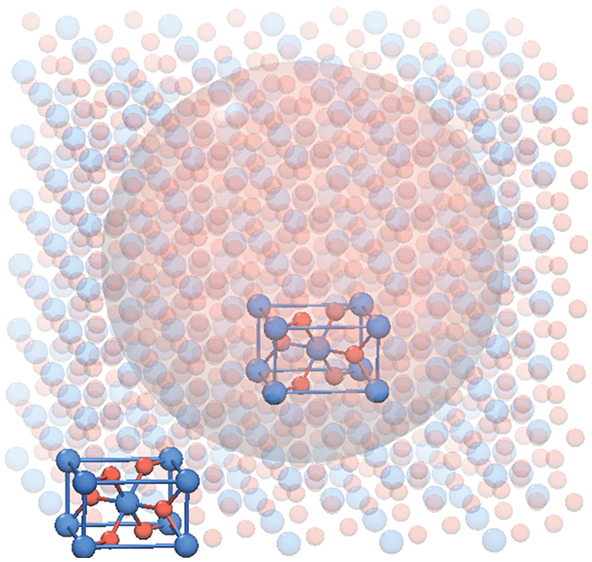

We study the effect of size on the vibrational modes and frequencies of nanoparticles, by applying a newly developed, robust, and efficient first-principles-based method that we present in outline. We focus on rutile TiO_2_, a technologically important material whose bulk exhibits a softening of a transverse acoustic mode close to , which becomes unstable with the application of negative pressure. We demonstrate that, under these conditions, nanoparticles above a critical size exhibit unstable localized modes and we calculate their characteristic localization length and decomposition with respect to bulk phonons. We propose that such localized soft modes could initiate coherent structural phase transformations in small nanoparticles above a critical size.

Much of the interest in nanomaterials derives from their size-dependent structural and functional properties, including their behavior under pressure. For example, size-dependent phase transformation pressures and pressure-induced shifts in Raman peak positions, optical absorption, and kinetic barriers, as well as a tendency to amorphization, have been observed by experimental and computational methods.^1−13^ The additional degrees of freedom associated with size, shape, and surfaces make pressure-induced phase transformations in nanoparticles much more complex and versatile in comparison to those in the bulk.

Bulk structural phase transformation pathways have long been classified as reconstructive or displacive, according to whether or not the breaking and making of interatomic bonds is involved. Reconstructive transformations tend to nucleate heterogeneously, whereas displacive transformations may involve homogeneous nucleation. An objective classification based upon symmetry^14^ distinguishes between two fundamental types (with a third type consisting of multistage combinations of the first two). In type I transformations, the lower-symmetry phase is related to the higher-symmetry phase by a unique distortion corresponding to a nontrivial irreducible representation of the higher-symmetry group. Here the lattice distortions and atomic displacements may therefore be correlated with the softening of one or more phonons of the corresponding symmetry. In type II transformations, the pathway between the two stable phases involves a common lower-symmetry intermediate.

In nanoparticles, structural changes may nucleate at or be frustrated by the presence of surfaces, whose influence is most simply measured by the surface to volume ratio that is size-dependent. This can lead to qualitative changes in the behavior of nanoparticles under pressure, e.g. from amorphization to recrystallization, at critical sizes as reported for metallic (e.g., silver^15^) and semiconductor nanoparticles (e.g., Si, CdSe, PbS, TiO_2_, and SnO_2_^16−21^). These structural changes may provide opportunities for applications that exploit improved properties; while certain desirable phases may only be stable in the bulk under unfavorable conditions for device operation, they may be more readily available as long-term metastable states in nanocrystals. For example, a method to generate self-sustained negative pressure in nanoparticles was developed and demonstrated for ferroelectric PbTiO_3_,^22^ which has the potential to be applied also to TiO_2_ nanoparticles and produce nanomaterials with advanced piezoelectric properties. To exploit the relationship between size and behavior under pressure, and thus tailor the properties of functional nanoparticles, a precise understanding of their interplay is crucial. Of particular interest is understanding what determines the critical size at which bulklike properties emerge.

Here we investigate this by focusing on a soft-mode-driven phase transformation in TiO_2_ rutile nanocrystals induced by isotropic tensile stress (negative pressure). Rutile-type TiO_2_, the most common phase of TiO_2_ with a wide range of technological applications,^23−28^ is an incipient ferroelectric whose dielectric constant increases with cooling until quantum fluctuations stabilize the ferroelectric instability at low temperature. The high dielectric constant is found to be caused by a low-frequency transverse optical A_2u_ mode at the Γ point.^29^ A particularly strong temperature and pressure dependence of the A_2u_ mode has been observed in experiments through neutron spectroscopy and Raman scattering.^30,31^ Density functional theory (DFT) calculations predict the softening of the A_2u_ mode under isotropic expansion linked to a ferroelectric phase transition.^32,33^ Separate from the A_2u_ mode, an anomalously soft transverse acoustic (TA) mode around  is found to be the first eigenmode whose frequency vanishes under isotropic tensile strain.^34^ Inelastic and diffuse X-ray scattering results confirm the existence of this soft TA mode,^35^ which is stabilized by anharmonic effects associated with the thermal expansion exploited to apply tension experimentally. The nature of the soft TA mode under negative pressure remains unclear.

To evaluate how these bulk soft modes might be linked to type I transformations in nanocrystals, we developed a procedure for constructing the dynamical matrix of a nanoparticle of arbitrary shape and size from the results of first-principles calculations of the bulk material, which is illustrated schematically in Figure 1. The five steps of the procedure are as follows.

**Figure 1:**
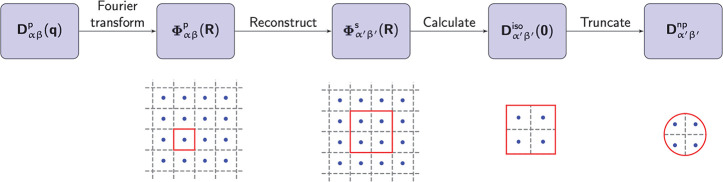
Flowchart of the five-step procedure used to construct the dynamical matrix of a nanoparticle of arbitrary shape and size.

Step 1. The dynamical matrix of the bulk primitive cell, **D**_αβ_^p^(**q**), is calculated on a discrete *N* = *N*_1_ × N_2_ × *N*_3_ grid of **q** points in the first Brillouin zone using density functional perturbation theory (DFPT).^36^

Step 2. The corresponding interatomic force constant matrix of the bulk, Φ_αβ_^p^(**R**), is obtained by discrete Fourier transformation of **D**_αβ_^p^(**q**) onto the corresponding *N*_1_ × *N*_2_ × *N*_3_ grid of real-space lattice vectors **R**:
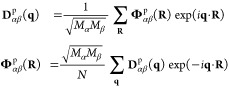
Here α and β denote atoms in the primitive unit cell of mass *M*_α_ and *M*_β_. Φ_αβ_^p^(**R**=**R***m*–**R***n*) describes the Hessian of the total energy with respect to the displacements of atoms α and β in primitive cells *m* and *n* (located at **R***m* and **R***n*), respectively. The matrix elements become small when *m* and *n* are sufficiently far apart.

Step 3. For a sufficiently large real-space grid **R**, the information in Φ_αβ_^p^(**R**) can be used to construct the force constant matrix of a *N*_1_ × *N*_2_ × *N*_3_ supercell Φ_α′β′_^s^(**R**′), where α′ and β′ denote atoms in the supercell and **R**′ refers to a supercell vector.

Step 4. An inverse Fourier transformation of Φ_α′β′_^s^(**R**′) results in the dynamical matrix of the *N*_1_ × *N*_2_ × *N*_3_ supercell ***D***_α′β′_^s^(**q**′) . Since only **q**′ = **0** and **R**′ = **0** are meaningful for an isolated nanoparticle, the dynamical matrix of the nanoparticle consisting of the atoms within a single supercell, **D**_α′β′_^iso^, is
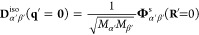
(see the Supporting Information for details).

Step 5. A spherical or arbitrarily shaped nanoparticle is then carved out of the single supercell nanoparticle by identifying atoms outside the desired surface and deleting the corresponding columns and rows in **D**_α′β′_^iso^, thus creating the dynamical matrix of the nanoparticle **D**_α′β′_^np^. In this case, we effectively delete the bonds that cross the surface of the nanoparticle, corresponding to freestanding boundary conditions. We can also implement fixed-surface boundary conditions, where displacement of the surface atoms is forbidden. With the size and shape of the nanoparticle and the boundary conditions being determined, the vibrational frequencies and normal modes are calculated by diagonalizing **D**_α′β′_^np^.

For the TiO_2_ systems studied here, our DFT calculations are performed with the CASTEP 18.1 code^37^ using a plane-wave basis set with an energy cutoff of 1300 eV. The local density approximation^38^ for exchange and correlation is chosen, since it has been shown to be suitable for rutile TiO_2_.^39^ Norm-conserving pseudopotentials are used: for Ti the 3s, 3p, 4s, and 3d orbitals are treated as valence states with the core radius *r*_c_ = 0.95 Å; for O, the 2s and 2p valence orbitals have *r*_c_ = 0.64 Å.^40^ The electronic sampling of the Brillouin zone is performed using a 4 × 4 × 8 Monkhorst–Pack grid.^34^ For the DFPT phonon calculations, a discrete grid of 15 × 15 × 25 wavevectors is used, which ensures that the corresponding supercell can accommodate a spherical nanoparticle with a maximum radius of 32 Å.

Since our treatment of the nanoparticle surface is very rudimentary, we validate the reliability of our method by comparing the frequencies obtained for the soft modes of interest with two different boundary conditions. For a spherical nanoparticle of radius 16 Å at −9 GPa there are five imaginary frequencies (tabulated in the Supporting Information), and the mean absolute deviation between the results for freestanding and fixed-surface boundary conditions is 0.01 cm^–1^, with relative errors all less than 1%. This demonstrates that our method is robust for these modes (typical of those of interest here), which we will later show are localized within the core of the nanoparticle with no significant contributions from the surface.

As the bulk transition pressure (here assumed negative) is approached, the phonon frequencies in a region of the Brillouin zone decrease. At the critical pressure, the frequency of a phonon at a **q** point away from Γ first vanishes. As the applied pressure is further decreased, the frequency of this phonon becomes imaginary and this instability could initiate a type I phase transition that is delocalized throughout the entire crystal. However, beyond the transition pressure, within a simulation of the undistorted and now mechanically unstable rutile structure, the region of the Brillouin zone with imaginary phonon frequencies extends beyond a single **q** point. Within the harmonic approximation, where all of these phonons are independent, we can imagine a linear combination of these soft modes that corresponds to a localized distortion in real space.^41^ If the size of the nanocrystal is large enough to accommodate the localized distortion, the nanocrystal may initially behave in a manner similar to that of its bulk counterpart, by transforming according to a coherent distortion without any nucleation at or influence from the surface.

Figure 2 shows the phonon frequencies of the bulk TA and A_2u_ modes under negative pressure, where the TA mode softens before the A_2u_ mode as the pressure decreases and thus is relevant to the initiation of any displacive phase transition. The displacement patterns of the soft TA phonons at  are mapped onto the Γ point of a 2 × 2 × 4 supercell. Because of the folding of the Brillouin zone, the soft TA mode is expanded into a 4-fold degenerate mode, one of which is shown in Figure 3. All four degenerate eigenmodes are dominated by displacements of Ti atoms along the ⟨110⟩ directions. The rutile structure is first distorted according to the [110] eigenmode and relaxed. The resulting structure is then distorted according to the [1̅10] eigenmode and again relaxed. Overall the rutile structure transforms from *P*4_2_/*mnm* to *I*2_1_2_1_2_1_. The inversion symmetry is lost, as expected for a ferroelectric phase transition.

**Figure 2:**
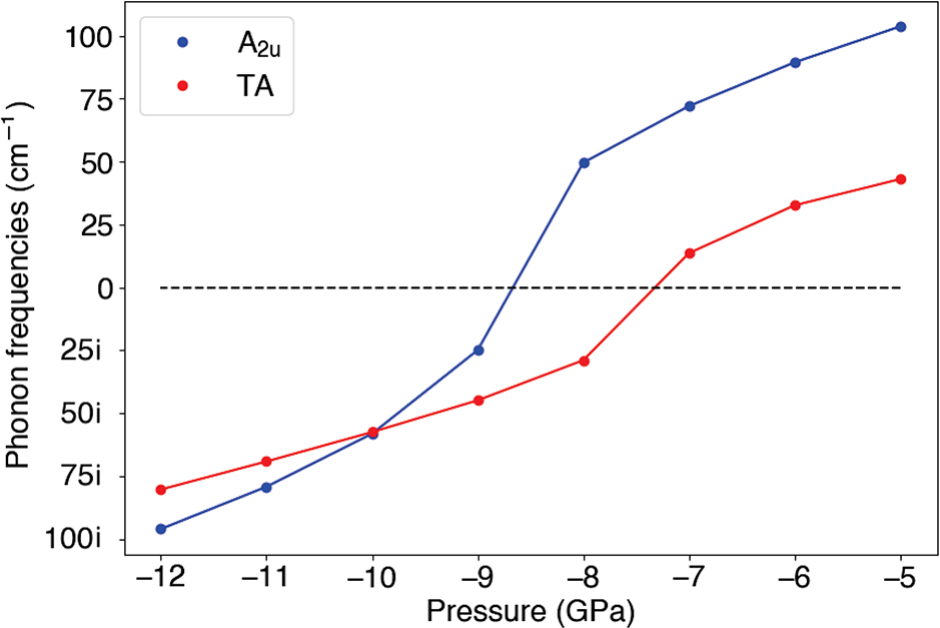
Phonon frequencies of the bulk soft TA and A_2u_ modes under negative pressure.

**Figure 3:**
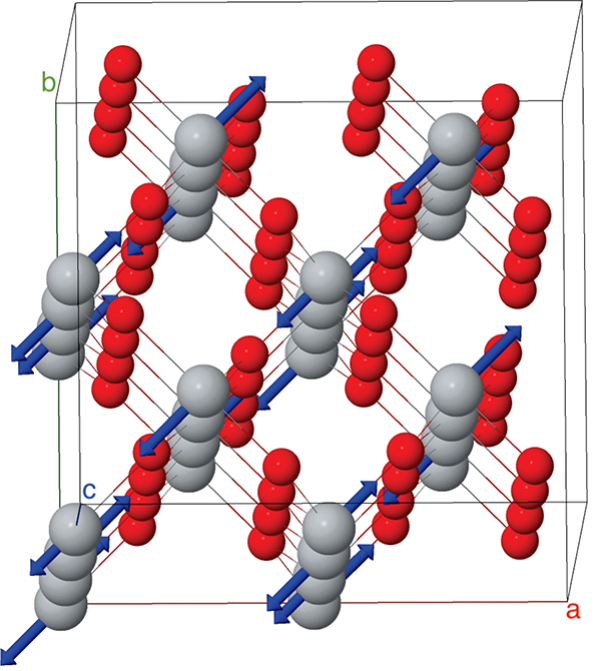
One of the 4-fold eigenmodes of the soft TA mode along the ⟨110⟩ directions. The others are shown in the Supporting Information.

Figure 4a shows the calculated size-dependent frequencies of the TA soft modes for a range of pressures between −7 and −9 GPa obtained by the procedure in Figure 1. The frequencies decrease as the sizes of these spherical nanoparticles increase, approaching the corresponding bulk limits and consistent with a confinement effect: the fit shown in Figure 4b is motivated by a simple model *P*_c_^np^(*r*_c_) = *P*_c_^b^ – *k*(*r*_c_ – *r*_0_)^−2^ with *P*_c_^b^ = −6.9 ± 0.3 GPa, *r*_0_ = 0.23 Å, and *k* = 373 GPa Å^2^. It is well-known that the transformation pressure of nanoparticles *P*_c_^np^ is greater (more negative in our case) than that of the bulk *P*_c_^b^.^42,43^ When the applied pressure is less negative than *P*_c_^b^, the frequencies of the soft modes remain positive, as is the case for −7 GPa in Figure 4a, and no phase transformation occurs for any size. In contrast, applying pressure more negative than *P*_c_^b^ (as for −9 GPa in Figure 4a) results in an unstable structure for the entire range of sizes for which a localized mode can be supported (in this case for radii above 15 Å).

**Figure 4:**
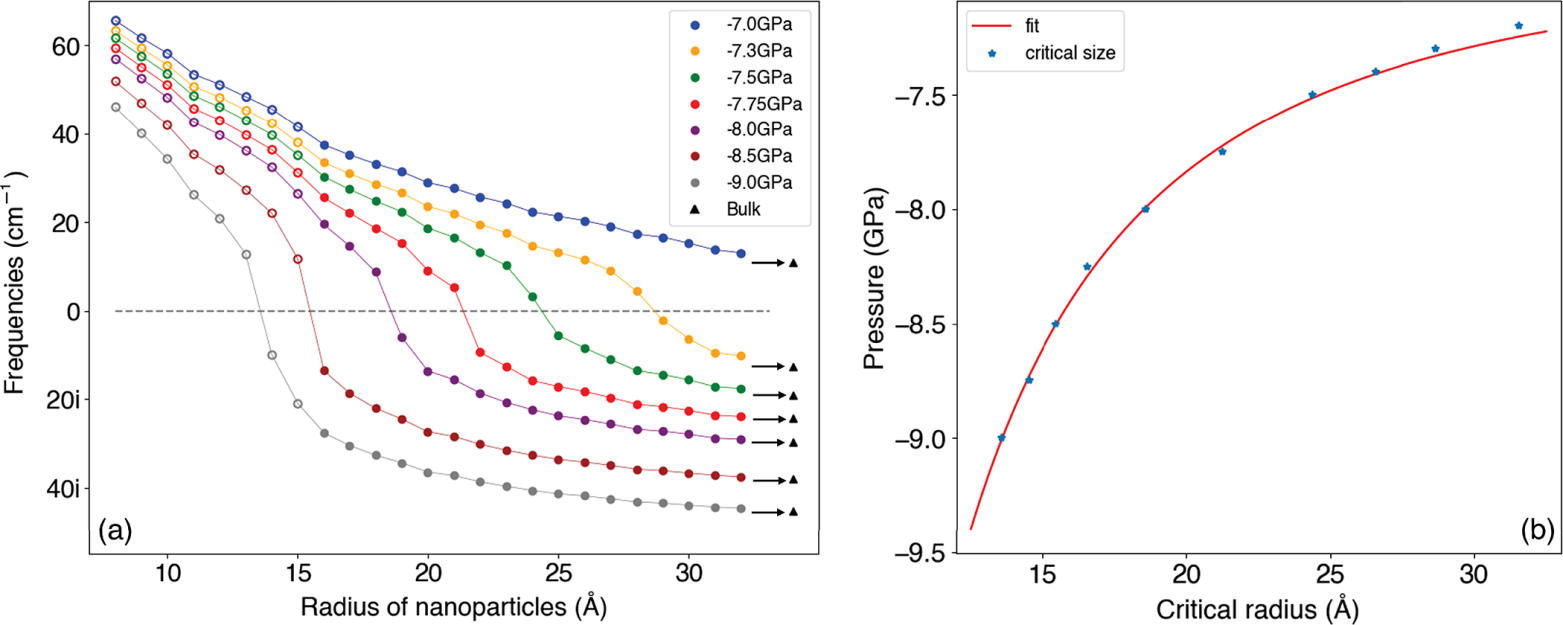
(a) Size dependence of soft mode frequencies at different pressures. The critical radius is estimated by the value at which the frequency vanishes. Filled symbols denote that the mode is localized (*L*_*n*_ > 0.7), whereas for open symbols the soft mode has significant weight at the surface (*L*_*n*_ < 0.7). The triangles indicate the bulk results. (b) Plot of negative pressure against critical radius. The fit is an inverse quadratic relationship expected from a confinement effect.

At intermediate pressures, e.g. –8 GPa in Figure 4a, the softest mode frequency changes from real to imaginary at a critical radius *r*_c_ of 19 Å. It should be noted that a negative pressure of −4.5 GPa has been achieved for PbTiO_3_ (bulk modulus 70 GPa)^22^ so that −8 GPa for TiO_2_ (bulk modulus 210 GPa) should be experimentally achievable.

To understand the evolution of the soft nanoparticle eigenmodes with size and pressure, we introduce two methods to analyze the nanoparticle eigenmodes. We first characterize the localization of nanoparticle eigenmode *n* by the quantity *L*_*n*_:
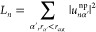
1where *u*_*nα*′_^np^ is the magnitude of the displacement of atom α′ in normalized mode *n* and *r*_α′_ is its distance from the center of the nanoparticle. The cutoff distance *r*_cut_ is chosen to be a fraction  of the nanoparticle radius, which divides it into core and surface regions of equal volume. For eigenmodes localized within the core of the nanoparticle, *L*_*n*_ approaches unity and decreases as the weight of the eigenmode close to the surface increases (*L*_*n*_ = 0.5 corresponds to equal weighting between the core and surface: i.e., delocalized over the whole nanoparticle). We also assess the nanoparticle eigenmode *n*, **u**_*n*_^np^, by decomposing it into a linear combination of the bulk phonons of the primitive cell extended to the supercell, **u**_*m***q**_^b^, by applying Bloch’s theorem.

The sum of the resulting expansion coefficients over all bulk phonon branches (labeled *m*)
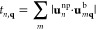
2can be used to analyze the distribution of bulk phonons contributing to a nanoparticle eigenmode across the Brillouin zone.

Figure 5 presents a series of bulk phonon frequency contour maps and calculated coefficients *t*_*n*,**q**_ for the softest mode (*n* = 1). Each panel represents the same slice of the Brillouin zone of the primitive cell spanned by the Γ–M () and Γ–Z  directions. Purple contours denote imaginary frequencies while the green/yellow contours are used for real frequencies. Comparing the top row (for −8 GPa) with the bottom row (−9 GPa) shows that the more negative pressure results in a greater region of the Brillouin zone where the softest mode is unstable.

**Figure 5:**
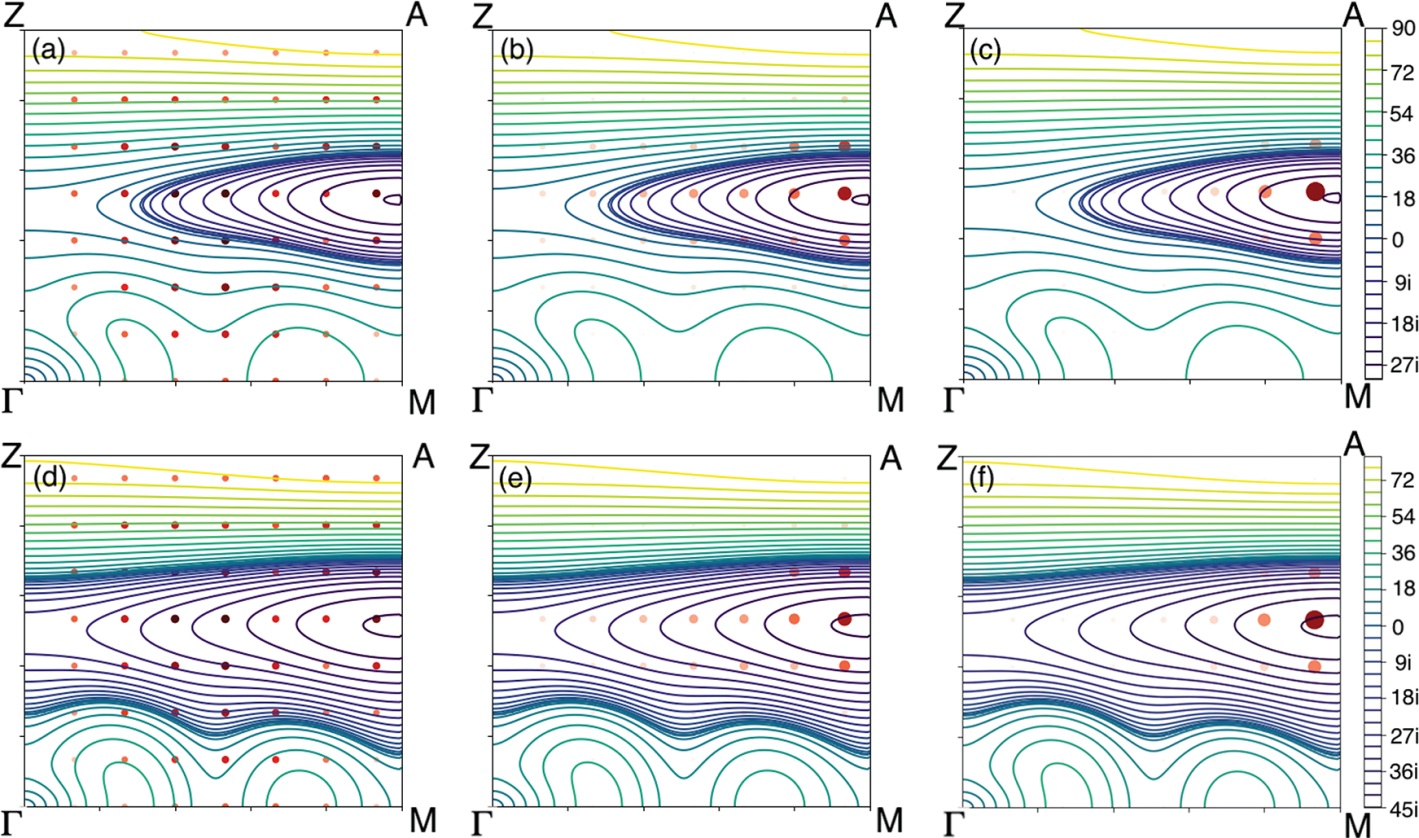
Contour maps representing the calculated softest bulk phonon frequencies across a slice of the Brillouin zone of the primitive cell at −8 GPa (a–c) and −9 GPa (d–f). These have been overlaid with the coefficients *t*_*n*=1,**q**_ for the discrete grid of **q** points for nanoparticles of radii 8, 16, and 24 Å for each column. The size and color of the red points represent the magnitude of the coefficients. The phonon frequencies for (a–c) are 57.0, 19.5 and 22.4*i* cm^–1^ and for (d)–(f) are 46.1, 27.6*i*, 40.6*i* cm^–1^ respectively.

To compare the relationship between the phonon modes of the bulk, **u**_*m***q**_^b^, and the normal modes of the nanoparticles, **u**_*n*_^np^, the expansion coefficient *t*_*n*=1,**q**_ is calculated from eq 2 for nanoparticles of radii 8, 16, and 24 Å and represented by red dots on a discrete mesh of **q** points (the size and color both denote magnitude). The coefficients for the smallest nanoparticle in the left-hand column, Figure 5a,d, are distributed throughout the whole Brillouin zone while for the largest nanoparticle, right-hand column, Figure 5c,f, they are mostly dominated by a small region where the phonon frequency is most strongly imaginary.

In Figure 5a (8 Å radius), the frequency of the eigenmode is 57.0 cm^–1^ and the localization *L*_1_ is 0.25, indicating that this nanoparticle is not large enough to accommodate a localized eigenmode and thus has significant weight on the surface atoms of the nanoparticle. The significant contribution from surface atoms leads to the distribution of the coefficients throughout the whole Brillouin zone, as is shown in Figure 5a. Our method is not designed to calculate the frequencies of such modes accurately.

In Figure 5c (24 Å radius), the frequency of the eigenmode is 22.4*i* cm^–1^ and the localization *L*_1_ is 0.95, reflecting that significant vibrational amplitudes are found only in the nanoparticle core. For this mode, the nanoparticle core behaves like the bulk. Such a localized distortion in real space corresponds to a region of the Brillouin zone (represented by the largest red points) that shrinks as the nanoparticle size increases, finally becoming a single **q** point as the nanoparticle approaches the bulk limit. As the size increases, the corresponding soft mode frequency decreases, approaching the bulk limit. This is consistent with the soft mode frequency curves in Figure 4a. The frequencies at −8 GPa decrease from 57.0 to 22.4*i* cm^–1^ as the radii of the nanoparticles increase from 8 to 24 Å.

We observe that the frequency of the eigenmode is gradually reduced from Figure 5a to Figure 5c and from Figure 5d to Figure 5f, which is due to the increasing contributions from the region of bulk eigenmodes with imaginary frequencies. This confinement effect on the frequencies has been shown in Figure 4, but Figure 5 clearly illustrates how this arises when a linear combination of bulk phonon modes **u**_*m***q**_^b^ constructs a localized distortion **u**_*n***q**_^np^. For sufficiently large nanoparticles, **u**_*n***q**_^np^ localizes inside the nanoparticle and corresponds to an imaginary frequency eigenmode of the nanoparticle. We note that the pattern of coefficients (red dots) is only subtly different between the two pressures shown in Figure 5: i.e. comparing Figure 5a with Figure 5d, Figure 5b with Figure 5e, or Figure 5c with Figure 5f. However, the bulk phonon contours differ significantly for these two pressures, resulting in the different behavior in the nanoparticle soft frequency versus size plots in Figure 4a.

Therefore, for nanoparticles that are sufficiently large to accommodate an unstable localized distortion, there is a potential pathway to a coherent transformation of the core structure that is initiated by the distortion. Such a phase transformation would occur spontaneously without an energy barrier, in contrast to crystal-to-crystal phase transformations with high energy barriers associated with bonding rearrangements in large nanoparticles or crystal-to-amorphous phase transformations with lower energy barriers in small nanoparticles.^44−47^ This potential pathway for coherent transformation of the core structure could be kinetically favorable relative to crystal-to-amorphous transformations for nanoparticles of intermediate sizes or could work in concert with other nucleation mechanisms, e.g., at surfaces, by propagating a rapid distortion of the core that results in distant nucleation events operating coherently. However, we would not expect to observe this mechanism in large nanoparticles due to the presence of defects.

In summary, we have confirmed the presence of a soft TA mode around  in bulk rutile TiO_2_ under negative pressure and we have identified the associated distortion and nearest stable structure that could be associated with a type I phase transformation. Furthermore, we have developed a DFT-based method to construct the dynamical matrix of a nanoparticle, which enables us to efficiently calculate the eigenmodes in nanoparticles comparable in size to those studied in experiments. Above a critical size and pressure, we observe a localization of soft modes, with the associated imaginary frequencies decreasing in magnitude as the size of the nanoparticle increases, approaching the bulk limit. By decomposing the eigenmodes of the nanoparticle in terms of the bulk eigenmodes, we find that nanoparticle eigenmodes localized in the nanoparticle core are a linear combination of the bulk eigenmodes from a small region of reciprocal space. In contrast, nanoparticle eigenmodes with significant contributions from atoms near the surface have contributions across the whole Brillouin zone. Therefore, it is possible that these localized soft modes may initiate coherent pressure-induced displacive phase transformations in nanoparticles above a critical size, large enough to accommodate them. This could lead to a qualitative change in behavior of small nanoparticles under pressure.

## Supporting Information Available

Definitions and conventions of Fourier transform, bulk soft mode eigenmodes and nanoparticle soft mode frequencies under different boundary conditions (PDF)
