# Supplementary material for: Localized Soft Vibrational Modes and Coherent Structural Phase Transformations in Rutile TiO2 Nanoparticles under Negative Pressure
Source: Nano Lett. 2022 Jul 7;22(14):5922–8. doi: 10.1021/acs.nanolett.2c01939 (PMC9335867; doi:10.1021/acs.nanolett.2c01939)
Supplement: Supplementary file 1 — nl2c01939_si_001.pdf [file nl2c01939_si_001.pdf]

Supporting Information for "Localized Soft Vibrational Modes and Coherent Structural Phase Transformations in Rutile TiO_2 Nanoparticles under Negative Pressure"

Kang Wang,[†] Carla Molteni,[‡] and Peter D. Haynes[*,†]

†*Imperial College London, Department of Materials, Exhibition Road, London SW7 2AZ, UK*

‡*King's College London, Department of Physics, Strand, London WC2R 2LS, UK*

E-mail: p.haynes@imperial.ac.uk

1. Definitions and conventions

The conventions for the discrete Fourier transforms between the dynamical matrix and the force constant matrix used in this work are as follows:

$$\boldsymbol{D}_{\alpha\beta}(\mathbf{q}) = \frac{1}{\sqrt{M_\alpha M_\beta}} \sum_{\mathbf{R}} \mathbf{\Phi}_{\alpha\beta}(\mathbf{R}) \exp(i\mathbf{q}\cdot\mathbf{R}), \tag{1}$$

$$\mathbf{\Phi}_{\alpha\beta}(\mathbf{R}) = \frac{\sqrt{M_\alpha M_\beta}}{N} \sum_{\mathbf{q}} \boldsymbol{D}_{\alpha\beta}(\mathbf{q}) \exp(-i\mathbf{q}\cdot\mathbf{R}), \tag{2}$$

where $\mathbf{R}$ are real space lattice vectors and $\mathbf{q}$ are reciprocal space wave vectors within the first Brillouin zone. The summations are carried out on regular meshes (with an odd number of points $N_i = 15, 15, 25$ in each direction $i = 1, 2, 3$ respectively, and $N = N_1 \times N_2 \times N_3$

in total) defined in terms of the primitive lattice vectors $\mathbf{R}_i$ and corresponding reciprocal space lattice parameters $\mathbf{G}_i$:

$$\mathbf{R} = m_1\mathbf{R}_1 + m_2\mathbf{R}_2 + m_3\mathbf{R}_3, \qquad m_i = 0, 1, \ldots, N_i - 1, \tag{3}$$

$$\mathbf{q} = \frac{l_1}{N_1}\mathbf{G}_1 + \frac{l_2}{N_2}\mathbf{G}_2 + \frac{l_3}{N_3}\mathbf{G}_3, \qquad l_i = -\tfrac{1}{2}\left(N_i - 1\right), \ldots, \tfrac{1}{2}\left(N_i - 1\right). \tag{4}$$

The following results are also used in the paper:

$$\boldsymbol{D}^{\text{iso}}_{\alpha'\beta'}(\mathbf{q}' = \mathbf{0}) = \frac{1}{\sqrt{M_{\alpha'}M_{\beta'}}}\boldsymbol{\Phi}^{\text{s}}_{\alpha'\beta'}(\mathbf{R}' = \mathbf{0}) \tag{5}$$

$$\boldsymbol{D}^{\text{iso}}_{\alpha\beta}(\mathbf{q}' = \mathbf{0}) = \frac{1}{\sqrt{M_{\alpha}M_{\beta}}}\sum_{\mathbf{R}'=\mathbf{0}}\boldsymbol{\Phi}^{\text{s}}_{\alpha\beta}(\mathbf{R}')\exp(i\mathbf{q}'\cdot\mathbf{R}') = \frac{1}{\sqrt{M_{\alpha}M_{\beta}}}\boldsymbol{\Phi}^{\text{s}}_{\alpha\beta}(\mathbf{0}) \tag{6}$$

2. Bulk soft mode eigenvectors

The fourfold degenerate soft transverse acoustic mode (TA) eigenvectors are shown in Fig. S1, of which one is shown in Fig. 3 of the paper.

3. Nanoparticle soft mode frequencies

Table S1 lists the lowest ten mode frequencies for a spherical nanoparticle of radius 16 Å under −9 GPa, with both freestanding and fixed-surface boundary conditions. The excellent agreement demonstrates that our method is robust for these modes, which are shown to be localized within the core of the nanoparticle in the paper.

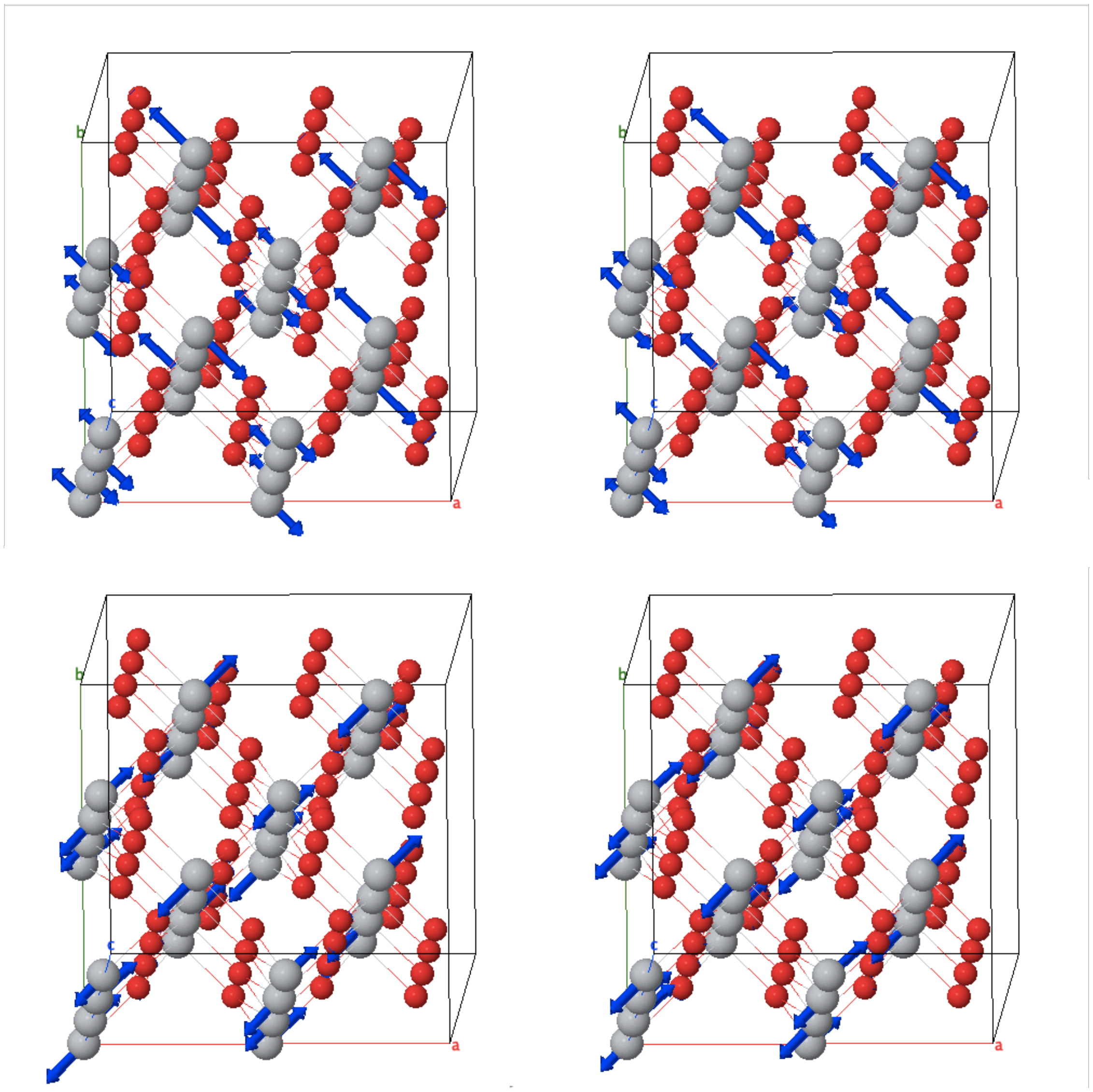

Figure S1: The fourfold degenerate eigenmodes of the soft TA mode along the $\langle 110 \rangle$ directions.

Table S1: The first ten vibrational frequencies for a spherical nanoparticle with radius 16 Å under −9 GPa with freestanding and fixed-surface boundary conditions. The three acoustic modes are not included.

Freestanding (cm^{-1})	Fixed-surface (cm^{-1})
$18.627i$	$18.629i$
$15.757i$	$15.763i$
$10.255i$	$10.255i$
$9.239i$	$9.241i$
$5.423i$	$5.475i$
7.571	7.577
8.620	8.623
12.114	12.114
12.300	12.300
14.032	14.025
